# Electroconvulsive therapy in the medical comorbidities context: A case report

**DOI:** 10.1192/j.eurpsy.2021.1826

**Published:** 2021-08-13

**Authors:** A. Sanz Giancola, M.D.C. Molina Lietor, M. Blanco Prieto, N. Freund Llovera, L. Nocete Navarro, I. Cuevas Iñiguez, C. Álvarez García

**Affiliations:** Psiquiatría, Hospital Universitario Príncipe de Asturias, Alcalá de Henares, Spain

**Keywords:** ECT, pacemaker, Depression, comorbidities

## Abstract

**Introduction:**

Electroconvulsive therapy (ECT) is today one of the main treatments available and used in psychiatry for serious mental illnesses. Eighty years after its introduction, the ECT procedure has evolved to become a safe option based on scientific evidence. Nowadays there are no absolute contraindications for ECT, regardless of the type of population and clinical situation.

**Objectives:**

To illustrate the electroconvulsive therapy in medical comorbidities context with a case report.

**Methods:**

Descriptive case study.

**Results:**

We present a 66 years old patient who suffers from a psychiatric decompensation with a diagnosis of major depressive disorder with psychotic symptoms. Due to her cardiological history (prolongation of the QT interval of possible psycopharmacological origin and a 2:1 AV block, that required the implantation of a definitive pacemaker) and partial response to psychotropic medication, the initiation of electroconvulsive therapy is proposed as the best alternative. The pacemaker was previously studied by cardiology for a very complete analysis before the procedure. It was recommended to convert it to fixed rate pacing by using a magnet. To do this, we placed it over the pacemaker during the technique. While waiting for a clinical improvement, no incidence has been produced during the sessions.

**Conclusions:**

ECT should not be postponed as a last resort. Numerous studies conclude that ECT is globally the treatment of choice (70-85% response) in severe depressive conditions, over and above antidepressant drugs. The incidence of relevant cardiac complications on ECT is relatively rare (0.9%). Regarding the use of pacemakers, electroconvulsive therapy represents an effective and safe option for the patient.

**Disclosure:**

No significant relationships.

